# Dynamic Change of Thyroid Hormones With Postmenstrual Age in Very Preterm Infants Born With Gestational Age <32 Weeks: A Multicenter Prospective Cohort Study

**DOI:** 10.3389/fendo.2020.585956

**Published:** 2021-03-30

**Authors:** Ranran Shi, Ming Zhang, Yao Chen, Meiying Han, Ping Xu, Min Li, Yanjie Ding, Xiaohui Zhang, Yan Kou, Haiyan Xu, Fangru Zong, Xinjian Liu, Hui Wang, Haiying He, Qiang Liu, Weikang Kong, Shiping Niu, Xia Li, Lei Huang, Qinghua Lu, Xiaofang Wang, Liping Deng, Zhenying Yang, Xiao Zhang, Rongrong Sun, Riming Zhao, Jing Shi, Fudong Peng, Xueming Sun, Guoying Zhao, Xinfeng Zhao, Yonghong Ge, Nan Zhang, Renxia Zhu, Jing Li, Haiyan Li, Huijuan Hao, Yonghui Yu

**Affiliations:** ^1^ Department of Neonatology, Shandong Provincial Hospital Affiliated to Shandong First Medical University, Jinan, China; ^2^ Department of Neonatology, Shandong Provincial Hospital, Cheeloo College of Medicine, Shandong University, Jinan, China; ^3^ Department of Neonatology, Liaocheng People’s Hospital, Liaocheng, China; ^4^ Department of Neonatology, Linyi Maternal and Child Health Hospital, Linyi, China; ^5^ Department of Neonatology, Yantai Yuhuangding Hospital, Yantai, China; ^6^ Department of Neonatology, Shandong Provincial Qianfoshan Hospital Affiliated to Shandong First Medical University, Jinan, China; ^7^ Department of Neonatology, Qilu Hospital, Cheeloo College of Medicine, Shandong University, Jinan, China; ^8^ Department of Neonatology, Hebei PetroChina Central Hospital, Langfang, China; ^9^ Department of Neonatology, Baogang Third Hospital of Hongci Group, Baotou, China; ^10^ Department of Neonatology, Linyi People’s Hospital, Linyi, China; ^11^ Department of Neonatology, Zibo Maternal and Child Care Hospital, Zibo, China; ^12^ Department of Neonatal Intensive Care Unit, Jinan Maternity and Child Care Hospital, Jinan, China; ^13^ Department of Neonatology, Shandong Maternal and Child Health Hospital, Jinan, China; ^14^ Department of Neonatology, Heze Municipal Hospital, Heze, China; ^15^ Department of Neonatology, Taian Maternal and Child Health Care Hospital, Tai’an, China; ^16^ Department of Neonatology, Dongying People’s Hospital, Dongying, China; ^17^ Department of Neonatology, Juxian People’s Hospital, Rizhao, China; ^18^ Department of Neonatology, Second People’s Hospital of Liaocheng, Liaocheng, China; ^19^ Department of Neonatology, Yidu Central Hospital of Weifang, Weifang, China; ^20^ Department of Neonatology, Binzhou Medical University Hospital, Binzhou, China; ^21^ Department of Neonatology, Maternal and Child Health Care Hospital of Zaozhuang, Zaozhuang, China; ^22^ Department of Neonatology, Liaocheng Dongchangfu Maternal and Child Health Care Hospital, Liaocheng, China; ^23^ Department of Neonatology, Jinan Central Hospital Affiliated to Shandong University, Jinan, China; ^24^ Department of Neonatology, Linzi District People’s Hospital, Zibo, China; ^25^ Department of Neonatology, The Second Affiliated Hospital of Shandong First Medical University, Jinan, China; ^26^ Department of Neonatology, Yantaishan Hospital, Yantai, China; ^27^ Department of Neonatology, Jinan Second Maternal and Child Health Hospital, Jinan, China

**Keywords:** thyrotropin, free thyroxine, preterm infant, postmenstrual age, congenital hypothyroidism

## Abstract

**Background:**

At present, the relationship between thyrotropin (TSH) and free thyroxine (FT4) in relation to postmenstrual age (PMA) in preterm infants is still unclear, and there is no reliable standard thyroid hormone reference ranges, resulting in different diagnostic criteria for congenital hypothyroidism been used by different newborn screening programs and different countries.

**Objectives:**

To investigate the relationship between TSH/FT4 and PMA in very preterm infants (VPIs) born with gestational age (GA) <32 weeks and to derive thyroid function reference charts based on PMA.

**Methods:**

A prospective cohort study was performed on VPIs born with GA<32 weeks and born in or transferred to the 27 neonatal intensive care units from January 1, 2019 to December 31, 2019. Serial TSH and FT4 values were measured at the end of each week during the first month after birth and also at PMA36 weeks, PMA40 weeks and at discharge, respectively. The 2.5th, 5th, 50th, 95th, and 97.5th percentiles of TSH and FT4 of different PMA groups were calculated to draw the percentile charts based on PMA.

**Results:**

1,093 preterm infants were included in this study. The percentile charts of TSH and FT4 levels based on PMA were drawn respectively, and the result indicated that the percentile charts of TSH values were gradually increased initially and then decreased with increasing PMA. The 97.5th percentile chart reached the peak at PMA30 weeks (17.38μIU/ml), and then decreased gradually, reaching the same level as full-term infants (9.07μIU/ml) at PMA38–40 weeks. The 2.5th percentile chart of FT4 was at its lowest point at PMA26–27 weeks (5.23pmol/L), then increased slowly with PMA and reached the same level as full-term infants at PMA38–40 weeks (10.87pmol/L). At PMA36 weeks, the reference intervals of the 2.5th to 97.5th percentiles of TSH and FT4 were 1.18–12.3μIU/ml and 8.59–25.98pmol/L, respectively.

**Conclusion:**

The percentile charts of TSH and FT4 in VPIs showed characteristic change with PMA. The results prompt that age-related cutoffs, instead of a single reference range, might be more useful to explain the thyroid function of VPIs. And repeated screening is necessary for preterm infants.

## Introduction

In recent years, the diagnosis of congenital hypothyroidism (CH) in preterm infants has increased gradually. Many newborn screening (NBS) programs provide repeated or serial neonatal screenings to improve the detection rate of CH in preterm infants (1–3). However, the detection methods used by different NBS programs are not the same, and the cutoffs are also different (4–7). Due to the lack of standardized and reliable reference cutoffs of thyroid hormones for preterm infants, the criteria used for CH diagnosis and drug replacement therapy in different countries around the world, including China, are different.

At present, the thyroid function of full-term infants has been fully studied and the corresponding reference ranges of thyrotropin (TSH) and free thyroxine (FT4) have been determined (8). However, there are only a few studies on the thyroid function of preterm infants, especially very preterm infants (VPIs) born with gestational age (GA) <32 weeks and the thresholds are not uniform. Infants with a venous TSH concentration >20mU/L with or without low FT4 are recommended for treatment by the 2014 European Society for Paediatric Endocrinology Consensus (9), however some studies reported missed CH cases with this threshold, especially in preterm infants (10). Therefore, a more reliable reference range is needed to evaluate the thyroid functions in preterm infants.

Previous studies have shown that the thyroid hormone levels of preterm infants vary with GA, birth weight (BW), postnatal age (PNA), and postmenstrual age (PMA) (1, 6, 11–17). Therefore, the application of the single reference range of thyroid hormones to preterm infants may produce false positive or false negative results (5, 10). Furthermore, most of the previous studies are single-center retrospective studies, with small sample sizes, thus researches on the relationships between TSH and FT4 and GA, PNA, and PMA through large preterm infant cohorts are helpful for clinicians to make a reasonable explanation for the thyroid function of preterm populations. Previous research had studied the relationship between TSH and PNA with large cohorts of preterm infants in Wisconsin and found that TSH levels vary with increasing PNA and this might explain the increased frequency of CH in preterm infants (18). But the TSH and FT4 values are also affected by GA, so it is not suitable to draw reference curves of TSH and FT4 based on PNA or GA alone. While PMA is the continuation of intrauterine GA, and it might be more valuable to establish the reference charts based on PMA. However, the relationship between TSH and FT4 and PMA is still under study.

To observe the trend of TSH and FT4 changes in relation to PMA, we conducted a large multicenter prospective cohort study based on the Shandong Neonatal Network (SNN), a regional multicenter neonatal clinical research database in China. The levels of TSH and FT4 in VPIs were detected dynamically after the first week of life to draw the dynamic percentile charts of TSH and FT4 based on PMA and to explore the changes of TSH and FT4 with PMA in very preterm infants, so as to provide a practical recommendation for standardized diagnosis and treatment of CH.

## Subjects and Methods

### Subjects

This study is a multicenter prospective cohort study. The data was collected from SNN, the regional multicenter neonatal clinical research database in China. Based on this database, the Multicenter Collaborative Group for Prognostic Evaluation of Very Low and Extremely Low Birth Weight Infants in Neonatal Intensive Care Units (NICUs) was established and a total of 27 NICUs from SNN were included in this study. The clinical data and TSH and FT4 values of all preterm infants born with GA<32 weeks who were born in or transferred to the 27 NICUs from January 1, 2019 to December 31, 2019 were collected prospectively. Subjects with the following conditions were excluded from the study, including incomplete clinical data, obvious congenital malformations or congenital chromosomal or genetic diseases, preterm infants who used levothyroxine or other drugs that might affect thyroid function, and the infants who died in hospital or transferred to other hospitals during hospitalization. The infants whose mother suffered from endocrine diseases or took drugs that might affect thyroid function, such as levothyroxine, were also be excluded.

In this study, 27 NICUs mainly used the primary TSH combined with FT4 method to evaluate the thyroid function of preterm infants and serial NBS was performed in all hospitalized preterm infants born with GA<32 weeks. In order to avoid the influence of TSH surge, the first screening was carried out at day 7 (1st week), and then the serum levels of TSH and FT4 were measured serially at day 14 (2nd week), day 21 (3rd week), day 28 (4th week), PMA36 weeks, and PMA40 weeks until discharge, and were recorded into the SNN database. If the PMA36 weeks were not reached or overlapped with the time of discharge, only the data at the time of discharge were filled into the database. The general clinical characteristics and all thyroid hormone values of the preterm infants were extracted from the database. After excluding the preterm infants who met the exclusion criteria, all the TSH and FT4 values of the included infants were collected.

### Grouping of the Subjects

The preterm infants were divided into groups according to the PMA at the time of blood collection. Since the minimum GA in this study was 25 weeks, PMA 26 week was taken as the first group. At the same time, because the sample sizes of PMA 26 week to 27 week and PMA 38 week to 40 week were small, they were combined into PMA 26-27 week group and PMA 38-40 week group, respectively. Other samples were divided into each PMA groups according to the PMA at the time of blood collection, such as PMA 28 week group, PMA 29 week group, until PMA 37 week group.

### Detection Methods

All NICUs collected the blood samples through arterial or venous puncture to measure the level of serum thyroid hormones in the morning and the blood volume was about 2ml for each test. Twenty two NICUs (22/27) used electrochemiluminescence immunoassay method to detect TSH and FT4 levels in undiluted serum samples and five NICUs (5/27) used chemiluminescence immunoassay method. All laboratories of the participating NICUs used standard serum samples to adjust the instruments regularly. The TSH levels were measured in all NICUs, but FT4 levels were not measured in two NICUs. For the two NICUs that did not measure FT4, the measurement methods of TSH used in the two NICUs were consistent with other NICUs. The measurement assays, manufactures of the instruments and agents, and the adult reference ranges of TSH and FT4 used in each participating center were listed in Appendix Table 1. 17/27 NICUs used the same Roche measurement instruments, agents and reference intervals. All data were filled in the SNN database in time and could be analyzed by clinicians.

### Ethical Approval

This study has been registered in the Chinese Clinical Trials Registry (Registration No. ChiCTR1900025234), and has been approved by the Ethics Committee of Shandong Provincial Hospital affiliated to Shandong First Medical University and Shandong University (LCYJ : NO.2019-132). The parents of all the subjects agreed to participate in this study and signed the informed consent form. The clinical data collected from each NICU is anonymous and encrypted, which conforms to the data protection law.

### Standard Biosecurity and Institutional Safety Procedures

We adhered to standard biosecurity and institutional safety procedures of Shandong Provincial Hospital affiliated to Shandong First Medical University and Shandong University.

### Statistics

The statistical analysis of this study was performed by SPSS 23.0 software. The 2.5th, 5th, 50th, 95th, and 97.5th percentiles of TSH and FT4 values were calculated for each PMA group and also the total samples, and the PMA-age related percentile charts were drawn. Differences between groups were analyzed by one-way ANOVA. Differences were regarded as significant when p<0.05.

## Results

**Figure 1:**
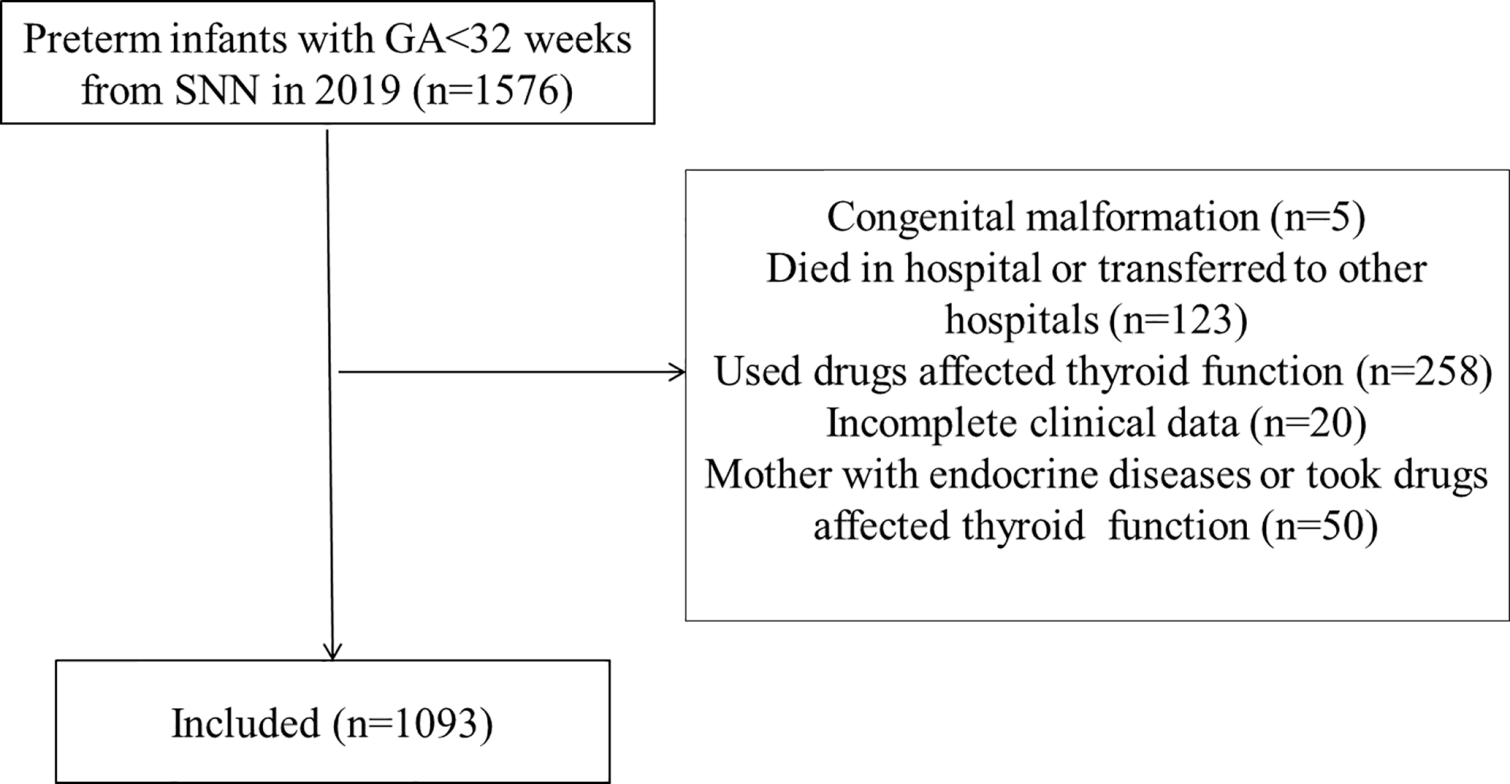
Inclusion flow chart.

A total of 1,576 preterm infants born with GA<32 weeks were included in this study and 1,093 cases were finally included after excluding 483 preterm infants who met the exclusion criteria (**Figure 1**). Among the cases, there were 106 infants born with GA<28 weeks and 987 infants born with GA 28–31^+6^ weeks. A total of 2,306 TSH values (about 2.1 TSH values per infant) and 2,194 FT4 values (about 2.0 FT4 values per infant) were collected. There were 582 males (53.2%) and 511 females (46.8%). The average born GA was 29.6 ± 1.4 weeks (median 30 weeks, range 25–31.6 weeks), and the average birth weight was 1,388.5g (median 1,380g, range 600–2,400g). The general characteristics of the study cohort included in the study were present in **Table 1**.

**Table 1:** General characteristics of the study cohort.

Total number of infants (n)	1,093
Birth GA, mean (SD), weeks	29.6 (1.4)
Birth weight, mean (SD), g	1388.5 (313.2)
Birth length, mean (SD), cm	38.8 (3.6)
Birth head circumference, mean (SD), cm	27.5 (2.3)
SGA/AGA/LGA infants, n (%)	
SGA	29 (2.7)
AGA	915 (83.7)
LGA	149 (13.6)
Sex, n (%)	
Male	582 (53.2)
Female	511 (46.8)
Singleton or multiplets, n (%)	
Singleton	849 (77.7)
Multiplets	244 (22.3)
Neonatal sepsis, n (%)	30 (2.7)
Moderate-severe BPD, n (%)	63 (5.8)
PS, n (%)	645 (59)
Dex after birth, n (%)	135 (12.4)
NEC of phase II and above, n (%)	16 (1.5)
IVH of phase III and above, n (%)	17 (1.6)
Birth Apgar score at 5,min<6, n (%)	66 (6.0)

AGA, appropriate for gestational age; SGA, small for gestational age; LGA, large for gestational age; BPD, bronchopulmonary dysplasia; PS, pulmonary surfactant; Dex, dexamethasone; NEC, necrotizing enterocolitis; IVH, intraventricular hemorrhage.

**Figure 2:**
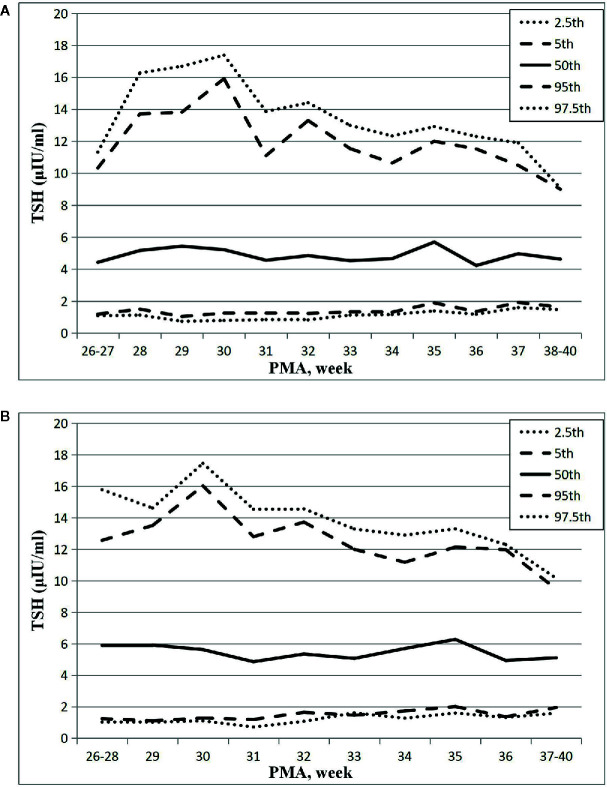
Reference chart of TSH based on PMA in preterm infants born with GA < 32 weeks. **(A, B)** represent the percentile charts of TSH based on PMA in total 27 NICUs and 17 NICUs using the same measurement assay, respectively.

The results showed that the 97.5th percentile of the TSH level was increased initially and then decreased from PMA26–27 weeks to PMA38–40 weeks. It reached a peak at the PMA30 weeks (17.38μIU/ml) and then gradually decreased to 12.3μIU/ml at PMA36 weeks and to 9.07μIU/ml at PMA38–40 weeks. The trend of the 95th percentile chart of TSH was basically consistent with that of the 97.5th percentile chart, which reached the highest point at PMA30 weeks (15.92μIU/ml). The reference interval from 2.5th to 97.5th percentile of the total cohort was 1.02–14.13μIU/ml (**Table 2**, **Figure 2A**). To analyze the impact of measurement methods on the reference charts, the 1,651 TSH values from the 17 NICUs using the same measurement method were also analyzed. Due to the small sample size of the PMA 26 to 28 weeks and PMA 37 to 40 weeks, the groups were combined into PMA26–28 weeks and PMA37–40 weeks, respectively. The trend of the 97.5th TSH percentile chart was consistent with the total sample results, increased to 17.46μIU/ml at PMA30 weeks and then decreased to 10.17μIU/ml at PMA37–40 weeks (**Figure 2B**).

**Table 2:** Percentiles of TSH values (μIU/ml) based on different PMA in preterm infants with GA < 32 weeks.

PMA, week	No. TSH values	TSH percentile				
		2.5th	5th	50th	95th	97.5th
26–27	24	1.09	1.19	4.43	10.33	11.32
28	52	1.13	1.50	5.17	13.71	16.27
29	104	0.74	1.04	5.44	13.82	16.69
30	204	0.79	1.25	5.22	15.92	17.38
31	328	0.85	1.25	4.56	11.10	13.86
32	469	0.83	1.23	4.85	13.30	14.41
33	378	1.13	1.33	4.53	11.54	12.99
34	287	1.16	1.31	4.66	10.64	12.33
35	142	1.39	1.89	5.70	12.00	12.92
36	229	1.18	1.35	4.23	11.52	12.30
37	39	1.60	1.92	4.97	10.49	11.90
38–40	50	1.47	1.63	4.63	9.00	9.07
Total	2,306	1.02	1.32	4.78	12.30	14.13

**Figure 3:**
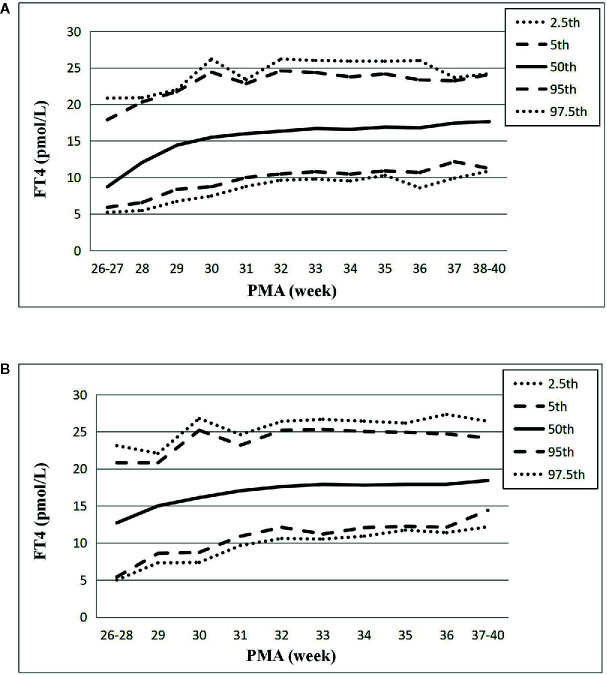
Reference chart of FT4 based on PMA in preterm infants born with GA < 32 weeks. **(A, B)** represent the percentile charts of FT4 based on PMA in total 27 NICUs and 17 NICUs using the same measurement assay, respectively.

The changes of the 2.5th, 5th, 50th, 95th, and 97.5th percentiles of FT4 from PMA26–27 weeks to PMA40 weeks were basically the same, showing a gradual upward trend. The 2.5th percentile chart of FT4 was at the lowest level at PMA26–27 weeks, about 5.23pmol/L (0.40ng/dl), and then increased slowly to 8.59pmol/L (0.66ng/dl) at PMA36 weeks and 10.87pmol/L (0.84ng/dl) at PMA38–40 weeks. The reference interval of percentile from 2.5th to 97.5th of the total cohort was 8.59–25.8pmol/L (0.66–1.98 ng/dl) (**Table 3**, **Figure 3A**). The 1651 FT4 values from the 17 NICUs using the same measurement method were also analyzed. The grouping of the FT4 values were the same as the TSH values. The trend of the 2.5th FT4 percentile chart was also consistent with the total sample results, increased gradually from 5.01pmol/L at PMA26–28 weeks to 12.07pmol/L (0.93ng/dl) at PMA38–40 weeks (**Figure 3B**).

**Table 3:** Percentiles of FT4 values (pmol/L) based on different PMA in preterm infants with GA < 32 weeks.

PMA, week	No. FT4 values	FT4 percentile				
		2.5th	5th	50th	95th	97.5th
26–27	22	5.23	5.91	8.72	17.90	20.85
28	50	5.47	6.57	12.05	20.33	20.93
29	98	6.73	8.38	14.41	21.76	22.01
30	195	7.46	8.73	15.50	24.40	26.18
31	305	8.78	10.00	16.00	22.85	23.40
32	438	9.63	10.48	16.33	24.59	26.20
33	354	9.77	10.78	16.70	24.35	26.01
34	280	9.52	10.46	16.59	23.77	25.91
35	141	10.29	10.90	16.89	24.17	25.90
36	226	8.59	10.68	16.80	23.35	25.98
37	38	9.90	12.16	17.45	23.23	23.66
38–40	47	10.87	11.23	17.65	24.09	24.19
Total	2,194	8.53	9.83	16.21	23.78	25.80

To evaluate the impact of PNA on the PMA-based reference charts, the TSH and FT4 values of PMA32 weeks with different PNA were analyzed because the sample size was large in PMA32 weeks. A total of 469 TSH values and 438 FT4 values were included and were divided into four groups according to the PNA, from the 1st week to the 4th week after birth. The 97.5th percentile values of TSH were ranged from 14.1 to 14.7μIU/ml, consistent with the 97.5th percentile values in the total PMA32 weeks group (14.41μIU/ml), and there were no significant differences in TSH values between adjacent PNA groups. Similarly, the 2.5th percentile values of FT4 were ranged from 8.95 to 10.62pmol/L, also consistent with the total sample results (9.63pmol/L), and no significant differences were found between adjacent PNA groups.

The 97.5th percentile of TSH at PMA36 weeks was 12.3μIU/ml, which was basically consistent with the ranges reported previously. **Table 4** showed the TSH reference ranges of preterm infants at PMA36 weeks reported previously.

**Table 4:** TSH reference ranges of preterm infants at PMA 36 weeks in previous studies.

GA, weeks	No. of cases (n)	TSH value (μIU/ml)	Percentile
<32	1,093	12.3	97.5th
22–27^18^	1,022	13	99th
28–31^18^	2,115	13	99th
25–36^19^	120	12	Single reference range
26–36^12^	247	12.53	Single reference range

GA, gestational age; TSH, thyrotropin

The above figures (12, 18, 19) represent the corresponding references in the Reference.

## Discussion

In this study, a multicenter prospective cohort study was conducted in 1,093 VPIs born with GA<32 weeks, and found that the percentile charts of TSH and FT4 changed dynamically with PMA. Twenty seven NICUs from SNN were included in this study, including tertiary and secondary comprehensive teaching hospitals and maternal and child health care hospitals, whose population distribution can represent the basic level of the Chinese population. Therefore, the data obtained in this study could be applicable to the population of China and even Asia.

The percentile charts of TSH level of VPIs varied with the increase of PMA in our study, and the result indicated that age-related TSH cutoffs might be more helpful for accurate diagnosis of CH in preterm infants. Previous studies also suggested that age-related cutoffs might be more useful and reliable (11, 18). However, some studies showed different results and suggested that all preterm infants could use a single TSH reference range (0.8–12μIU/ml) to assess their thyroid function (19). The differences of the results might be caused by different sample size, blood sample testing methods (such as whole blood tests by dried blood spots and serum tests), different GA, PMA, and BW of the preterm infants collected, statistical analysis methods and different definitions of healthy preterm infants. In this study, we conducted a prospective large sample size cohort study and found a characteristic relationship between TSH percentiles and PMA, which is more reliable and useful for defining the age-related cutoffs for preterm infants. At the same time, we also analyzed the levels of TSH at PMA36 weeks and the results are similar to the previous studies, ranging from 12 to 13μIU/ml (12, 18, 19).

In addition, the percentile charts of FT4 values were also increased slowly with PMA, which indicated that age-related cutoff might be more rational, and the 2.5th percentile chart of FT4 reached the full-term level at PMA38–40 weeks (10.87pmol/L), which is consistent with the previous reported level of full-term infants (12pmol/L) (12, 19). This change of FT4 might be related to the gradual development and maturation of hypothalamic pituitary thyroid (HPT) axis. For preterm infants born with GA<34 weeks, the transport of maternal thyroid hormone through the placenta might be suddenly interrupted prematurely during delivery, and the immaturity of the HPT axis in preterm infants can lead to the weakening or disappearance of T4 response to TSH surge after birth, leading to a lower level of FT4 in premature infants than in full-term infants. Therefore, with the increase of PMA, the HPT axis could be gradually developed and matured, and the level of FT4 could also be increased gradually (20). This phenomenon can explain the change of FT4 in our study. Thus, age-related cutoffs of FT4 might be more suitable for the evaluation of the thyroid function status in VPIs.

The adult reference intervals of FT4 of different NICUs using different measurement assays were quite different. To investigate the impact of the measurement assays on the results, the FT4 values of 17 NICUs using the same Roche assay were analyzed. The results were similar with the findings of the total samples and showed a more obvious upward trend with increasing PMA. The 97.5th percentile chart of TSH values of the 17 NICUs were also analyzed and the results were also consistent with trend of the total samples. Therefore, the impact of the measurement methods on the reference charts in this study was very small. While, the FT4 and TSH values of the other NICUs using other assays were unable to be analyzed due to the small sample size and the reference chart of FT4 and TSH of some assays might be different to a certain extent. To investigate the exact impact of each assay on the PMA-based FT4 and TSH reference intervals, studies with larger samples should be conducted in the future and the utilization of the reference charts should be validated in the hospitals using different assays.

To evaluate the influence of PNA on the PMA-based reference charts, the TSH and FT4 values of PMA32 weeks were divided into four groups according to PNA and one-way ANOVA was conducted to analyze the differences between PNA groups in this study. The result indicated that the TSH values and FT4 values of different PNA groups were consistent with the total sample results and no statistical differences were found between adjacent PNA groups. The results indicated that the reference intervals of TSH and FT4 based on the same PMA were equivalent for preterm infants of different PNA. Therefore, the PMA age-related cutoffs of TSH and FT4 are not affected by PNA. This result is consistent with previous results (19). Some researchers also proposed different results and considered that the TSH and FT4 values of different PNA might be different (18). However, most of the previous studies focused on the change of TSH values based on PNA alone, and the impact of GA was not analyzed. Moreover, the characteristics of the population and the sample size of the studies were different, which might also bring about differences in the results. Hence, the PMA-based reference charts need to be further validated in different populations and studies.

Previous studies have also suggested that some non-thyroid diseases such as bronchopulmonary dysplasia (BPD), severe sepsis, intraventricular hemorrhage (IVH), necrotizing enterocolitis (NEC) in newborns, and the use of some drugs might affect thyroid hormone levels (21, 22). In addition, small for gestational age (SGA), Apgar score at birth, and the use of drugs such as glucocorticoids after birth might also affect thyroid function, so that some studies excluded the preterm infants with these diseases to define healthy preterm infants (6, 12, 19, 21–24). However, the impact of these factors on thyroid hormone levels is still unclear, and most preterm infants will face these diseases after birth, so that excluding these infants might affect the representativeness of the population. Iodine status may also affect the thyroid function (25), but Shandong is a coastal province and iodine is widely added into salt according to the requirements of the Chinese Food Administration. Therefore, iodine deficiency is rare in the preterm infants included in this study. In the following study, we should further explore the effects of these factors on thyroid hormone levels in preterm infants.

In this study, a prospective multicenter observational cohort study was conducted on VPIs born with GA<32 weeks. Dynamic changes of TSH and FT4 with PMA were found and the reference charts from PMA26–27 weeks to PMA38–40 weeks were established. Based on the percentile charts, we recommend using age-related percentile charts of TSH and FT4 levels based on PMA, rather than a single reference range, as the cutoff values for the assessment of the thyroid function in VPIs. Serial screening in all VPIs is necessary to reevaluate the thyroid status, especially at PMA38–40 weeks, because at this point, the TSH and FT4 levels will reach the same level as the full-term infants. For extremely preterm infants (EPIs) born with GA<28 weeks, the thyroid function should also be retested at PMA30 weeks, as the TSH levels might reach the peak at this time point.

There are also some limitations in our study. Some studies have shown that there is a significant difference in thyroid function status between EPIs with GA<28 weeks and those with GA >28 weeks (18). However, due to lack of data of EPIs in this study, it is impossible to group the infants according to GA. Therefore, in the following study, with the gradual increase in the amounts of cases, we will further group and analyze the thyroid function according to the stratification of GA. In addition, preterm infants with some non-thyroid diseases are not excluded, and the measurement methods used in different NICUs were also different, and the effect of these diseases and specific assays on the thyroid function of preterm infants is still unclear, thus, in the following study, we should further analyze the impact of these factors on the thyroid function of preterm infants. At the same time, all infants treated with levothyroxine tablets were excluded from this study, and the criteria used for diagnosis and treatment of premature infants with TSH in the range of 6–20μIU/ml is different in each NICU, which might result in excluding of these preterm infants from the study, thus bias the research results. Therefore, in the future, we need to carry out a prospective case control study or randomized controlled trials (RCT) on this cohort to further explore the outcome of their thyroid function and the level of long-term neurodevelopment between the treated group and the untreated group. Moreover, all data included in this study are from hospitalized preterm infants and the follow-up data after discharge have not yet been tracked, therefore, regular follow-up is needed for discharged preterm infants to further extend the reference charts and clarify the trend of thyroid development in preterm infants.

## Conclusion

This study shows characteristic dynamic changes between TSH and FT4 levels and PMA. We recommend using the age-related percentile charts of TSH and FT4 values, instead of a single reference range, as the cutoffs for the assessment of thyroid function in VPIs. This study may provide a basis for the construction of age-related reference range of thyroid hormones in preterm infants and might be of great value for the standardized diagnosis and treatment of CH in preterm infants.

### Special Definitions

Gestational age (GA): calculated by the time of the last menstruation or by ultrasound in the first trimester.

Postmenstrual age (PMA): gestational age +postnatal age.

SGA/AGA/LGA is defined according to the Fenton intrauterine growth curve (26).

Congenital hypothyroidism (CH): 1 week after birth, if the serum TSH >20μIU/ml with or without low FT4 levels, levothyroxine is administered immediately, and then the level of TSH should be dynamically retested to adjust the dose. If serum TSH level is between 6 and 20μIU/ml, the thyroid function should be retested one week later. This definition is referred to the 2014 European Society of Pediatric Endocrinology guidelines for CH (9).

Neonatal septicemia: it is diagnosed by the following simultaneous presence: 1) clinical manifestations of newborn infection (according to ≥1 items): respiratory distress, apnea; tachycardia or bradycardia; systemic hypotension or hypoperfusion; hypothermia or fever (T>38.5°C or <36°C); convulsion, hypotonia, irritability or lethargy; feeding intolerance or intestinal obstruction. 2) Abnormal non-specific infection index (according to ≥2 items): WBC<5×10^9/L, or WBC increased (>20×109/L); CRP≥10mg/L; PLT ≤ 100×10^9/L; PCT>2ng/ml. 3) Positive blood or cerebrospinal fluid culture (27).

The staging of BPD (28), IVH (29), and NEC (30) are referred to the corresponding guidelines.

## Data Availability Statement

The datasets generated and analyzed in the current study are not readily publicly available because the data is collected by the Multicenter Collaboration Group of Shandong Neonatal Network (SNN), and our relevant research has not been published. Requests to access the datasets could be directed to the corresponding author (YY, alice20402@126.com).

## Ethics Statement

The studies involving human participants were reviewed and approved by Ethics Committee of Shandong Provincial Hospital affiliated to Shandong First Medical University and Shandong University (LCYJ: NO.2019-132). Written informed consent to participate in this study was provided by the participants’ legal guardian/next of kin.

## Author Contributions

YY designed the study and revised the manuscript content. RSh collected and analyzed the data, and drafted the manuscript. Other authors collected and submitted the data into Shandong Neonatal Network database. All authors contributed to the article and approved the submitted version.

## Funding

This work has been supported in part by Grant from National Research Center for Assisted Reproductive Technology and Reproductive Genetics.

## Conflict of Interest

The authors declare that the research is conducted in the absence of any commercial or financial relationships that could be construed as a potential conflict of interest.
