# Supplementary material for: Dynamic Change of Thyroid Hormones With Postmenstrual Age in Very Preterm Infants Born With Gestational Age <32 Weeks: A Multicenter Prospective Cohort Study
Source: Front Endocrinol (Lausanne). 2021 Mar 30;11:585956. doi: 10.3389/fendo.2020.585956 (PMC8043151; doi:10.3389/fendo.2020.585956)
Supplement: Supplementary file 1 [file DataSheet_1.pdf]

APPENDIX TABLE 1. THE SPECIFIC ASSAYS AND THE ADULT REFERENCE INTERVALS OF TSH AND FT4 USED IN THE PARTICIPATING CENTERS

Collaborative Hospital	Measurement Methods	Manufactures of the Instruments	Manufactures of the Reagents	TSH （μIU/ml）	FT4 （pmol/L）
Shandong Provincial Hospital-East Part	Electrochemiluminescence immunoassay method	Roche	Roche	0.27-4.20	12.00-22.00
Shandong Provincial Hospital-Central Part	Electrochemiluminescence immunoassay method	Roche	Roche	0.27-4.20	12.00-22.00
Liaocheng People's Hospital	Chemiluminescence immunoassay method	Beckman DXI800	Beckman	0.56-5.91	7.98-16.02
Linyi Maternal and Child Health Hospital	Electrochemiluminescence immunoassay method	Roche	Roche	0.27-4.20	12.00-22.00
Yantai Yuhuangding Hospital	Electrochemiluminescence immunoassay method	Roche	Roche	0.27-4.20	12.00-22.00
Shandong Provincial Qianfoshan Hospital	Electrochemiluminescence immunoassay method	Roche	Roche	0.27-4.20	12.00-22.00
Qilu Hospital, Cheeloo College of Medicine	Chemiluminescence immunoassay method	Abbott	Abbott	0.35-4.94	9.01—19.05
Hebei PetroChina Central Hospital	Chemiluminescence immunoassay method	Beckman DXI800	Briliance	0.49-4.90	7.64-16.03
Baogang Third Hospital of Hongci Group	Electrochemiluminescence immunoassay method	Roche	Roche	0.27-4.20	12.00-22.00
Linyi People's Hospital	Electrochemiluminescence immunoassay method	Siemens	Siemens	0.55-4.78	11.50-22.70
Zibo Maternal and Child Care Hospital	Electrochemiluminescence immunoassay method	Roche	Roche	0.27-4.20	12.00-22.00
Jinan Maternity and Child Care Hospital	Electrochemiluminescence immunoassay method	Beckman DXI800	Yingsheng	0.34-5.60	9.98-28.30
Shandong Maternal and Child Health Hospital	Electrochemiluminescence immunoassay method	Siemens	Siemens	0.55-4.78	11.50-22.70
Heze Municipal Hospital	Electrochemiluminescence immunoassay method	Roche	Mindray	0.50-7.30	6.50-18.20
Taian Maternal and Child Health Care Hospital	Electrochemiluminescence immunoassay method	Roche	Roche	0.27-4.20	12.00-22.00
Dongying People's Hospital	Electrochemiluminescence immunoassay method	Roche	Roche	0.27-4.20	12.00-22.00
Juxian People's Hospital	Chemiluminescence immunoassay method	Abbott	Abbott	0.35-4.94	9.01-19.05
Second People's Hospital of Liaocheng	Chemiluminescence immunoassay method	Beckman DXI800	Beckman	0.56-5.91	——
Yidu Central Hospital of Weifang	Electrochemiluminescence immunoassay method	Roche	Roche	0.27-4.20	12.00-22.00
Binzhou Medical University Hospital	Electrochemiluminescence immunoassay method	Roche	Roche	0.27-4.20	12.00-22.00
Maternal and Child Health Care Hospital of Zaozhuang	Electrochemiluminescence immunoassay method	Roche	Roche	0.27-4.20	12.00-22.00
Liaocheng Dongchangfu Maternal and Child Health Care Hospital	Electrochemiluminescence immunoassay method	Roche	Roche	0.27-4.20	12.00-22.00
Jinan Central Hospital	Electrochemiluminescence immunoassay method	Roche	Roche	0.27-4.20	12.00-22.00
Linzi District People's Hospital	Electrochemiluminescence immunoassay method	Roche	Roche	0.27-4.22	12.00-22.00
Yantaishan Hospital	Electrochemiluminescence immunoassay method	Beckman DXI800	Beckman	0.56-5.91	——
The Second Affiliated Hospital of Shandong First Medical University	Electrochemiluminescence immunoassay method	Roche	Roche	0.27-4.20	12.00-22.00
Jinan Second Maternal and Child Health Hospita	Electrochemiluminescence immunoassay method	Roche	Roche	0.27-4.20	12.00-22.00
